# Causal association between obstructive sleep apnea and amyotrophic lateral sclerosis: a Mendelian randomization study

**DOI:** 10.3389/fnagi.2024.1357070

**Published:** 2024-05-16

**Authors:** Rongrong Du, Yahui Zhu, Peng Chen, Mao Li, Ying Zhang, Xusheng Huang

**Affiliations:** ^1^Department of Neurology, The First Medical Center, Chinese PLA General Hospital, Beijing, China; ^2^School of Medicine, Nankai University, Tianjin, China; ^3^Medical School of Chinese PLA, Beijing, China; ^4^Department of General Surgery and Institute of General Surgery, The First Medical Center of Chinese PLA General Hospital, Beijing, China; ^5^The Fourth of the Health Department, The Second Medical Center, Chinese PLA General Hospital, Beijing, China

**Keywords:** obstructive sleep apnea, amyotrophic lateral sclerosis, causality, genetic association, Mendelian randomization

## Abstract

**Background:**

Obstructive sleep apnea (OSA) had a high prevalence in the population. Whether OSA increases the risk of amyotrophic lateral sclerosis (ALS) is unknown. Our aim was to clarify this issue using two-sample Mendelian randomization (MR) analysis in a large cohort.

**Methods:**

Two-sample MR was used to evaluate the potential causality between OSA and ALS by selecting single-nucleotide polymorphisms (SNPs) as instrumental variables (IVs) from genome-wide association studies (GWAS). The inverse-variance weighted (IVW) method was chosen as the primary method to estimate causal association. Weighted median, weighted mode and simple mode methods were used as sensitivity analyses to ensure the robustness of the results.

**Results:**

In MR analysis, IVW mode showed genetic liability to OSA was found to be significantly associated with a higher ALS risk (OR, 1.220; 95% confidence interval, 1.031–1.443; *p* = 0.021). No evidence of heterogeneity and horizontal pleiotropy were suggested.

**Conclusion:**

We found potential evidence for a causal effect of OSA on an increased risk of ALS.

## 1 Introduction

Obstructive sleep apnea (OSA) is a common sleep disorder that results in decreased hemoglobin oxygen saturation and disrupted sleep due to repeated apnea. Loud snoring, insomnia and daytime sleepiness are the main clinical manifestations. The overall prevalence of OSA in the general population is estimated to be 9–38% (Senaratna et al., 2017), varying with BMI, sex, age, and apnea-hypopnea index definitions used for diagnosis. OSA is about twofold to threefold more prevalent among men (5.3–49.7%) than women (1.2–23.4%) (Heinzer et al., 2015).

However, it is important to highlight that despite increasing public awareness and more cases being diagnosed, 80% of individuals with moderate or severe OSA remain undiagnosed, including a large proportion of ethnic and other minorities, older adults, and women (Billings et al., 2021). Failure to promptly address this condition may lead to various mechanisms inherent to OSA, such as intermittent hypoxia, sleep structure disruption, and heightened oxidative stress, thereby elevating the likelihood of severe comorbidities (Giampa et al., 2023). For example, a meta-analysis of cross-sectional and longitudinal studies has demonstrated that untreated OSA was associated with an increased risk of hypertension in the general population (Hou et al., 2018). In addition, previous research has demonstrated that OSA potentially amplifies the risk of stroke (Redline et al., 2010), mild cognitive impairment, and Alzheimer’s disease (AD) (Andrade et al., 2018; Ou et al., 2024), while also correlating with heightened Parkinson’s disease (PD) severity (Elfil et al., 2021; Tang et al., 2024).

Amyotrophic lateral sclerosis (ALS) is a fatal neurodegenerative disease of the central nervous system, mainly caused by degeneration and loss of upper and lower motor neurons. The common clinical manifestations were progressive muscle weakness, muscular atrophy and dyspnea. At present, the exact pathogenesis is not clear, and there is no effective treatment. The prognosis of ALS patients is poor, and the median survival in ALS is only 2 to 4 years (Feldman et al., 2022). Previous studies have found that the mean survival in ALS patients with an obstructive apnea/hypopnea index (AHIo) ≥5 was significantly shorter than in ALS without OSA (*p* = 0.0237), suggesting that OSA may contribute to disease progression in ALS (Quaranta et al., 2017). OSA appears to be more common in ALS patients (Boentert et al., 2018; Boentert, 2019). A meta-analysis revealed significant reductions in sleep efficiency, total sleep time, and increases in oxygen desaturation index, and apnea hypopnea index in ALS patients compared with controls (Zhang et al., 2023). Previous studies have suggested a high prevalence of OSA in ALS patients, and OSA could predict a shorter survival of ALS. These studies might suggest a bidirectional effect between OSA and ALS, but whether OSA can increase the risk of ALS is unclear.

Although previous epidemiological studies have linked OSA to central nervous system (CNS) disorders, such as, OSA may be an important risk factor for stroke, ALS patients with an OSA phenotype were characterized by a worse prognosis, and OSA might potentiate neuropathological and clinical progression of AD (Yaggi et al., 2005; Quaranta et al., 2017; Andrade et al., 2018), it is not entirely clear whether OSA is associated with CNS disease or whether OSA increases the risk of CNS disease.

Mendelian randomization (MR) studies are causal studies that use genetic variants to assess the association between risk factors and outcomes (Davies et al., 2018). Because genetic variants are randomly assigned at birth, unconfounded investigations can be conducted and reverse causality in observational studies can be avoided. Given that the association between OSA and ALS risk is unclear, the aim of this study was to use MR study to assess the effect of OSA on ALS risk.

## 2 Materials and methods

Ethical review and approval were waived for this study, due to this study used summary data from GWAS and did not involve individual data. All studies that contributed data to this analysis were approved by the relevant institutional review board. Patient consent was waived due to this study used summary data from GWAS and did not involve individual data.

### 2.1 Data source

This MR study utilized pooled data from the OSA genome-wide association study (GWAS), with study participants from the FinnGen study (Strausz et al., 2021). The GWAS comprised 16,761 OSA patients and 201,194 controls. National health registries were employed for the identification of OSA cases, using ICD codes (ICD-9: 3472A, obstructive sleep apnea; ICD-10: G47.3, sleep apnea), as provided by the Finnish National Hospital Discharge Registry and the Cause of Death Registry, OSA was identified.

For ALS GWAS Iacoangeli et al. (2020) meta-analyzed the aggregated statistics of two ALS GWAS: an ALS study that included more than 80,000 individuals of European descent (Nicolas et al., 2018) and a Chinese ALS study with more than 4,000 individuals (Benyamin et al., 2017). The meta-analysis included a total of 84,694 individuals, including 22,040 cases and 62,654 controls, and a total of 5,356,204 SNPs.

### 2.2 The selection of instrumental variable

**Figure 1:**
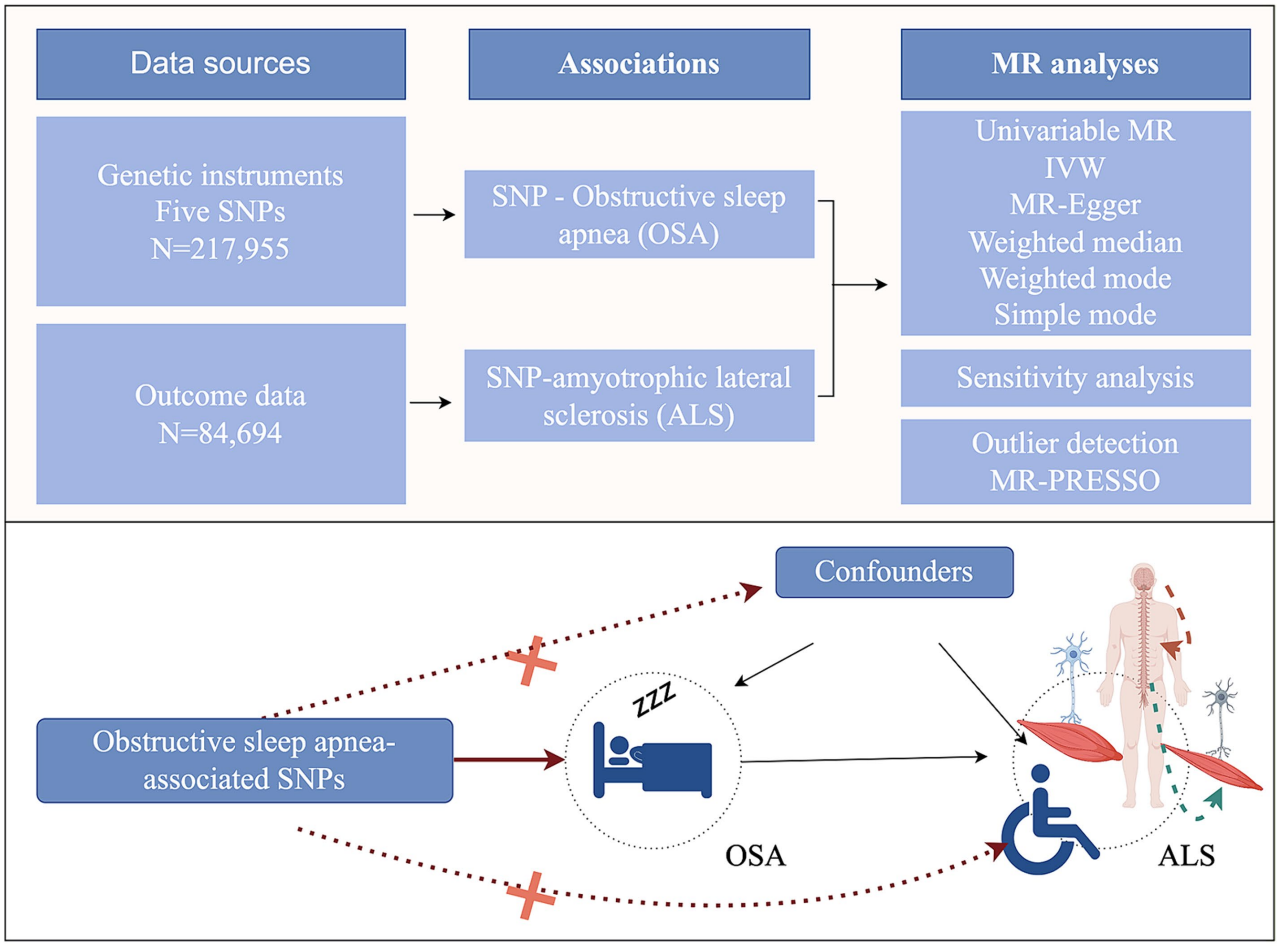
Study design of the two-sample Mendelian randomization for the effect of genetically predicted obstructive sleep apnea on amyotrophic lateral sclerosis (by Figdraw). MR, Mendelian randomization; IVW, inverse-variance weighted; SNPs, single-nucleotide polymorphisms.

By employing genetic variants that exhibit a robust association with exposure as instrumental variables (IVs), MR subsequently examined the causal relationship between genetic predisposition to exposure and the desired outcomes. The MR analysis should adhere to three hypotheses: (1) Genetic variants must display a significant correlation with the exposure factors; (2) Genetic variants should not be linked to potential confounding variables; (3) Genetic variants should solely influence the outcome through the pathway of exposure (Figure 1). We used OSA GWAS from FinnGen Study.  This GWAS identified 5 distinct genetic loci associated with OSA (*p* < 5.0 × 10^−8^). All these 5 SNPs were in different genomic regions and not in linkage disequilibrium (*r*^2^ < 0.20). And all these 5 SNPs had a high imputation quality (INFO >0.9) (Strausz et al., 2021). Thus, five genetic variants associated with OSA were identified. After that, we obtained the corresponding SNPs from the outcome (ALS) GWAS summary data and made the data harmonization.

### 2.3 Statistical analysis

To evaluate the impact of genetically-predicted OSA on ALS for each SNP, we employed the Wald ratio (Hemani et al., 2018). The inverse variance weighted (IVW) model was used as the primary MR analysis to evaluate the aggregate effect of multiple SNPs (Bowden et al., 2019). The IVW method essentially assumes a zero intercept and performs a weighted regression of the SNP-exposure effects with the SNP-outcome effects.

In the context of MR study, it is imperative that three assumptions are fulfilled to ensure the validity of the MR method. Firstly, it is crucial that genetic variants exhibit a significant association with the risk factors (exposure). Consequently, to minimize any possible weak IV bias, the strength of the IV was assessed using the F-statistic, denoted as F = β^2^/se^2^ (where β represents the effect size of the SNP on the exposure and Se represents its corresponding standard error). A higher F-statistic corresponded to a smaller bias. And if F-statistic > 10, it indicates that the study had sufficient strength (Burgess et al., 2011). The second assumption is deemed valid only if the genetic variants do not exhibit any association with confounding factors influencing the relationship between OSA and ALS. That is, there is no horizontal pleiotropy. The MR-Egger intercept (Burgess and Thompson, 2017) was used to study the influence of potential horizontal pleiotropy. MR-Egger method tests and accounts for the presence of unbalanced pleiotropy by introducing a parameter for this bias and incorporating outline information estimates of causative effects from multiple individual variants. The MR-PRESSO method mainly detects horizontal pleoitropy by using residual sum (Verbanck et al., 2018). In MR-PRESSO method, it attempts to reduce pleoitropy in the estimate of the causal effect by removing outliers that contribute to the pleoitropy disproportionately more than expected. Heterogeneity was assessed using Cochrane’s Q values, and if heterogeneity was present, the multiplicative random effects model was preferred.

We then performed sensitivity analyses, in which we assessed the consistency of MR results by using different methods established under different hypotheses to determine the robustness of our study. Weighted median, weighted mode and simple mode methods are used initially. The IVW approach requires that the pleiotropic effect of the genetic variants should be independent of exposure. Therefore, if the genetic variants do not conform to the hypothesis, the results of the weighted median method can offer a reliable effect estimation, despite up to 50% of the genetic variantions not aligning with the corresponding presumption. Furthermore, by assuming that the most common value of the bias in the estimation of Wald rations is zero, a weighted model-based approach can potentially yield coherent results without relying on measurement error assumptions that are not applicable. In addition, scatter plots were employed to illustrate effect estimates derived from different MR approaches.

In this study, we used R (version 4.1.2) and the “TwoSampleMR” and “MRPRESSO” packages (version 0.5.6) for analyses. In all of the above analyses, *p* < 0.05 indicates statistical significance.

## 3 Results

As part of this study, we evaluated the causal relationship between OSA and ALS. Table 1 shows the statistical data of the five SNPs selected as valid instrumental variables. The F statistic of each SNP was above the empirical threshold of 10.

**Table 1:** Extracted SNPs for the exposure OSA based on a genome-wide significance threshold of 5E-08.

SNP	A1	A2	EAF	BETA	SE	P	F statistic
rs9937053	G	A	0.43	0.102	0.013	4.32E-16	61.6
rs10507084	C	T	0.179	0.109	0.016	2.80E-11	46.4
rs4837016	G	A	0.466	−0.071	0.013	1.53E-08	29.8
rs185932673	C	T	0.003	0.624	0.112	2.44E-08	31.0
rs10928560	C	T	0.195	−0.088	0.016	2.80E-08	30.3

OSA, obstructive sleep apnea; SNP, single-nucleotide polymorphism; A1, effect allele; A2, non-effect allele; EAF, Effect allele frequency; BETA, beta estimate for the association of SNP with exposure; SE, standard error of the Beta; *P*, two-sided *P*-value from the meta-analysis for exposure.

**Figure 2:**
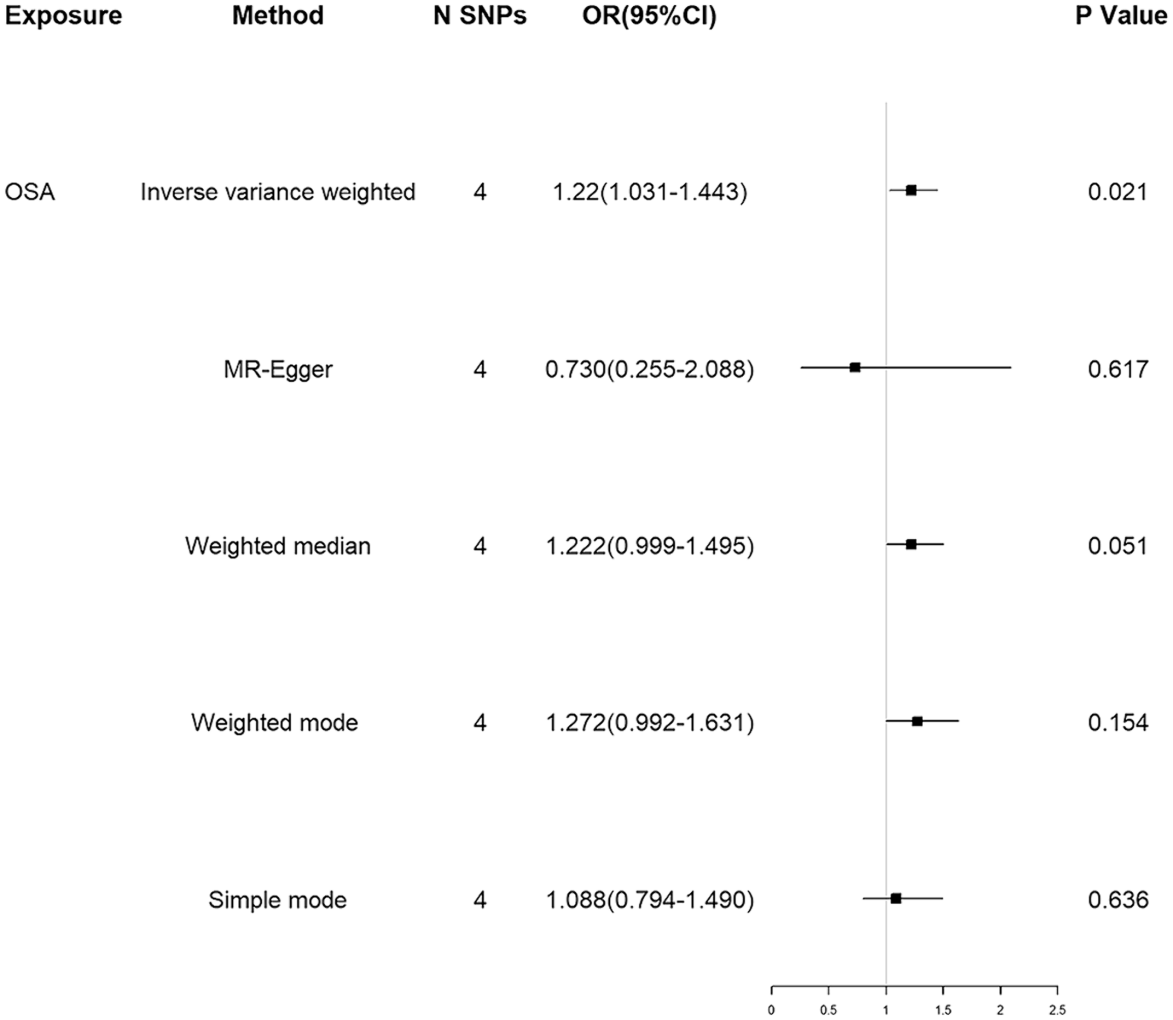
MR associations between genetically determined OSA and the risk of ALS. MR, Mendelian randomization; SNP, single nucleotide polymorphism; OR, odds ratio; CI, confidence interval; OSA, obstructive sleep apnea; ALS, amyotrophic lateral sclerosis.

**Figure 3:**
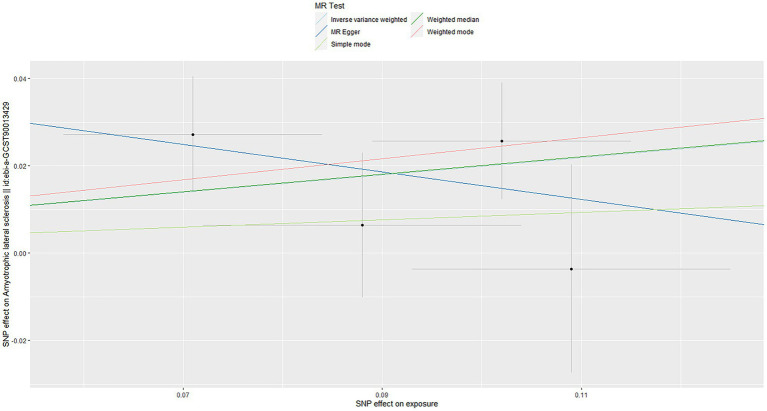
Scatter plots of genetic associations of OSA with ALS risk. The slopes of each line represent the causal association for each method. OSA, obstructive sleep apnea; ALS, amyotrophic lateral sclerosis.

Using the IVW method, our results suggested that genetically predicted OSA increased the risk of ALS [OR = 1.220 (1.031–1.443)] (Figure 2). Moreover, weighted median, weighted mode and simple mode also showed the same trend (Figure 2). Figure 3 showed the scatter plots of effect estimates derived from different MR methods.

No indications of heterogeneity were found in the causal effect estimates derived from the MR Egger and IVW analyses (all *p*-values <0.05, Table 2). The MR-PRESSO global test (*p* = 0.522) and MR Egger intercept (intercept = 0.047, *p* = 0.434, Table 2) suggested no horizontal pleiotropy for instrumental variables. The absence of heterogeneity and horizontal pleiotropy in this study suggested the robustness of the findings.

**Table 2:** Heterogeneity and pleiotropy tests of instrument effects.

Exposure	N SNPs	Heterogeneity analysis				Pleiotropy analysis			
		Method	Q	Degree of freedom	*P*	Method	Egger intercept	SE	*P*
OSA	4	MR Egger	1.76	2	0.416	MR Egger intercept	0.047	0.048	0.434
		IVW	2.70	3	0.441	MR-PRESSO Global test			0.522

SNP, single nucleotide polymorphism; MR, Mendelian randomization; IVW, inverse variance weighted; OSA, obstructive sleep apnea; SE, standard error.

## 4 Discussion

Using MR method, our results suggested that genetically predicted OSA increased the risk of ALS, suggesting the need for timely OSA intervention to reduce the risk of ALS in individuals at high risk of ALS.

In observational studies, previous studies have shown that ALS patients with OSA have shorter survival, suggesting that OSA might promote disease progression in ALS (Quaranta et al., 2017). In addition, a meta-analysis of 11 studies revealed that OSA could hasten the severity of cognitive decline and exacerbate motor symptoms in individuals with PD (Elfil et al., 2021). Another study also indicated an increased risk of developing PD in those with OSA (Chen et al., 2015). Our findings, utilizing the MR method, suggest that OSA is associated with an augmented risk of developing ALS.

OSA could potentially increase the risk of developing ALS, as suggested by previous studies. Xie et al. (2013) found that natural sleep or anesthesia in live mice led to a 60% increase in interstitial space, which could result in a significant increase in convective exchange of cerebrospinal fluid with interstitial fluid, potentially contributing to ALS pathology. The increased flow of interstitial fluid during sleep, in turn, improves the clearancee of β-amyloid. Consequently, sleep is a fundamental part of the processes involved in the removal of brain toxic metabolites (Xie et al., 2013). However, since OSA can cause sleep awakening and sleep fragmentation, affecting overall sleep quality, it is possible to increase the accumulation of toxic proteins in the brain of OSA patients, which may increase the risk of ALS.

Secondly, intermittent hypoxia (IH) is the main characteristic of OSA. It is well known that the brain is more sensitive to hypoxia than other organs, requiring more energy and oxygen consumption. The results of clinical and animal studies suggest that IH induced by OSA can lead to structural neuronal injury and dysfunction in the CNS, and oxidative stress and inflammatory damage are the pathophysiological basis (Liu et al., 2020). Accumulating evidence supports the idea that IH may induce ROS production, oxidative stress overactivation, and inflammatory damage in the CNS, leading to neuronal apoptosis and/or necrosis (Almendros et al., 2011). Similarly, in the mouse model of ALS, chronic intermittent hypoxia increases motor neuron death, neuromuscular weakness, and possibly cognitive dysfunction in mice (Kim et al., 2013). The generation of oxidative stress and the activation of inflammatory pathways may be related to it (Kim et al., 2013). In addition, previous studies have suggested that oxidative stress and inflammation are involved in the development of ALS (Teleanu et al., 2022). Therefore, we thought that intermittent hypoxia (due to OSA) promotes oxidative stress and inflammation of neurons (also characteristic of ALS), thereby further increasing ALS risk.

Thirdly, the respiratory force of respiratory collapse during OSA is associated with increased intrathoracic and intracranial pressures, and hemodynamic disturbance (Konecny et al., 2014; Wszedybyl-Winklewska et al., 2017). These studies hypothesized that this pressure impedes the flow of brain metabolites from interstitial fluid (ISF) to cerebrospinal fluid (CSF) via the glymphatic system (Ju et al., 2016), resulting in an increased accumulation of abnormal proteins in ISF and a significant decrease in neuro-derived proteins in CSF. Ju et al. (2016) elucidated the mechanism of neuro-derived proteins reduction in CSF and abnormal accumulation in ISF in patients with severe OSA. This suggests that the glymphatic clearing process is impaired in OSA patients. The decrease of abnormal SOD1 protein and TDP-43 protein clearance is considered to be one of the pathogenesis, which might also be another way for OSA to increase the risk of ALS.

Our study empolyed MR to assess the causal relationship between genetically predicted OSA and ALS risk. The study’s main advantage is its inclusion of a large number of participants in ALS GWAS. Furthermore, the MR design prevents reverse causality bias and balances potential confounders, since genetic variants are not associated with other common comorbidities, such as obesity, stroke, and high blood pressure, which can affect the results in observational studies. However, some limitations need to be noted in future studies: First, because OSA is a binary exposure, possible selection bias due to underdiagnosis cannot be well assessed. Second, because individual-level data were not available, potential bias due to medication status in ALS patients was not considered. Third, genetically predicted exposures frequently encompass enduring impacts, potentially intensified in magnitude, thereby rendering MR estimates distinct from clinical trial outcomes. Moreover, interventions targeting pertinent factors may not necessarily yield analogous clinical advantages as observed in MR studies. Fourth, the unavailability of stratified GWAS data for OSA severity hindered further exploration of the association between OSA severity and clinical characteristics. Fifth, the limited number of SNPs tested might affect the robustness of the results, and we will conduct further analysis in the future if larger OSA GWAS data and more SNPs are available. Sixth, we used the MR-Egger intercept and the MR-PRESSO method to evaluate the pleiotropy in this study. Although the results suggested that no horizontal pleiotropy for instrumental variables in this study, it cannot be completely ruled out whether the SNPs associated with exposure can have an impact on ALS risk through other ways. This is one of the limitations of this study. Finally, because this study focused primarily on participants of European descent, the results may not be extrapolated to populations of other ethnicities and will need to be further validated in other populations in the future.

While our study has limitations, it is the first to use MR method to examine the relationship between OSA and ALS. This study undertook a genetic perspective to evaluate the causal relationship between the two variables, suggesting that direct intervention with OSA may be beneficial in reducing the risk of ALS. It remains unclear how OSA raises ALS risk, and further studies are needed to discover how OSA affects neurodegenerative diseases in the brain.

## 5 Conclusion

Based on the results of the MR analysis, genetically predicted OSA leads to an increased risk of ALS. However, the mechanism by which OSA might increase the risk of ALS is currently unclear, and further study is needed to clarify it in the future.

## Data availability statement

OSA GWAS summary data are publicly available and can be downloaded from GWAS catalog (https://www.ebi.ac.uk/gwas/) and ALS GWAS summary data are publicly available and can be downloaded from https://gwas.mrcieu.ac.uk/.

## Ethics statement

Ethical approval was not required for the study involving humans in accordance with the local legislation and institutional requirements. Written informed consent to participate in this study was not required from the participants or the participants’ legal guardians/next of kin in accordance with the national legislation and the institutional requirements.

## Author contributions

RD: Conceptualization, Data curation, Writing – original draft. YaZ: Data curation, Formal analysis, Methodology, Writing – review & editing. PC: Data curation, Formal analysis, Writing – review & editing. ML: Data curation, Formal analysis, Writing – review & editing. YiZ: Investigation, Validation, Writing – review & editing. XH: Conceptualization, Project administration, Supervision, Validation, Writing – review & editing.
